# Cryo-EM Structure of HER2-trastuzumab-pertuzumab complex

**DOI:** 10.1371/journal.pone.0216095

**Published:** 2019-05-01

**Authors:** Yue Hao, Xinchao Yu, Yonghong Bai, Helen J. McBride, Xin Huang

**Affiliations:** 1 Department of Molecular Engineering, Amgen Inc., Cambridge, MA, United States of America; 2 Amgen Postdoctoral Fellow Program, Cambridge, MA, United States of America; 3 Department of Molecular Engineering, Amgen Inc., South San Francisco, CA, United States of America; 4 Biosimilars, Amgen Inc., One Amgen Center Drive, Thousand Oaks, CA, United States of America; University of Washington, UNITED STATES

## Abstract

Trastuzumab and pertuzumab are monoclonal antibodies that bind to distinct subdomains of the extracellular domain of human epidermal growth factor receptor 2 (HER2). Adding these monoclonal antibodies to the treatment regimen of HER2-positive breast cancer has changed the paradigm for treatment in that form of cancer. Synergistic activity has been observed with the combination of these two antibodies leading to hypotheses regarding the mechanism(s) and to the development of bispecific antibodies to maximize the clinical effect further. Although the individual crystal structures of HER2-trastuzumab and HER2-pertuzumab revealed the distinct binding sites and provided the structural basis for their anti-tumor activities, detailed structural information on the HER2-trastuzumab-pertuzumab complex has been elusive. Here we present the cryo-EM structure of HER2-trastuzumab-pertuzumab at 4.36 Å resolution. Comparison with the binary complexes reveals no cooperative interaction between trastuzumab and pertuzumab, and provides key insights into the design of novel, high-avidity bispecific molecules with potentially greater clinical efficacy.

## Introduction

Human epidermal growth factor receptors (HER) are a family of 4 transmembrane tyrosine kinase receptors that can dimerize with one another and mediate cell growth, differentiation, and survival.[1] In total, ten different homo- and heterodimers are formed by four HER receptors, allowing for integration of complex biological signaling events. Over-expression of HER2 has been shown to correlate with aggressive tumors, making it a key target for development of anti-cancer agents.[2, 3]

Structural studies have shown that the extracellular domain (ECD) of the HER family of receptors is composed of four subdomains (I-IV), and that the ECD can only exist in two forms: a tethered form and an extended form. In the tethered form the ECD is unable to mediate dimerization, due to interactions between subdomain II and subdomain IV.[4] However, in the extended form, the dimerization elements of the receptor are fully exposed allowing dimerization and signaling. HER2 is unique in that it exists in a constitutively extended form due to stabilization through direct interactions between subdomains I and III, explaining both why HER2 is a preferred binding partner for other HER family members and contributing to its importance in tumor development. [4, 5]

Trastuzumab, a therapeutic antibody targeting subdomain IV of the HER2 ECD, results in inhibition of HER2-mediated mitogenic signaling and a reduction in cell proliferation by blocking homodimerization of the protein.[6] The use of trastuzumab in HER2-positive cancer has transformed the treatment paradigm, but resistance has posed a serious limitation on its overall impact, provoking investigation into complementary therapies against this target.[7] The development of pertuzumab, a monoclonal antibody targeting subdomain II of the HER2 ECD is one such treatment, designed to block heterodimerization as well as homodimerization to more completely inhibit HER2 signaling.[4] The use of pertuzumab in combination with trastuzumab and docetaxel chemotherapy has improved clinical outcomes, justifying the use of this approach.[8] There are multiple hypotheses for how such synergy is produced including in silico models showing that enhanced binding affinity towards the HER2 molecule may result from cooperative interactions between the two antibodies.[9] If true, this would influence the design of improved bi-specific molecules for the treatment of HER2-positive cancers. Thus, it is of interest to better understand the structure and dynamics of a ternary complex including the HER2 ECD, trastuzumab and pertuzumab to inform on the design of novel therapeutic candidates. This paper describes results from cryo-EM structural studies on the ternary HER2-trastuzumab-pertuzumab complex and discusses the implications of the results on the design of novel therapeutics.

## Results and discussion

**Fig 1:**
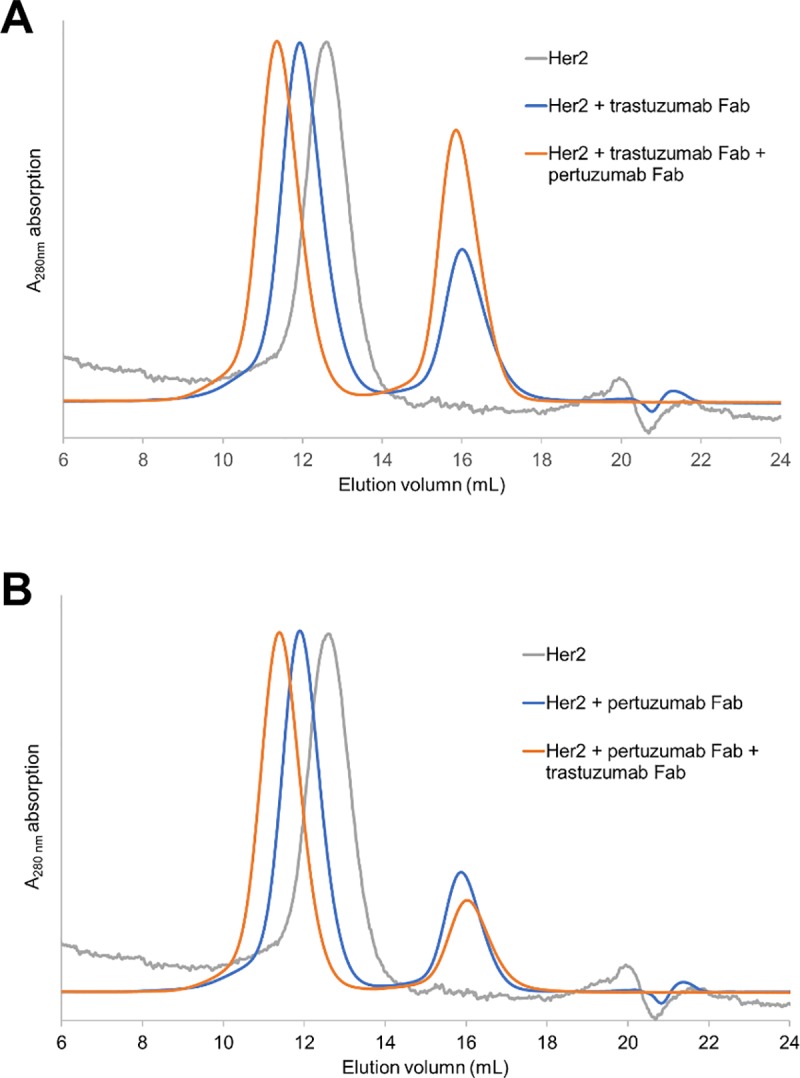
SEC profiles showing the formation of HER2-trastuzumab-pertuzumab ternary complex. A. HER2 is first complexed with trastuzumab Fab and the binary complex elutes earlier than HER2 alone. The purified complex is further complexed with pertuzumab Fab and the ternary complex elutes earlier than the binary complex. The UV absorption is normalized. B. HER2 is first complexed with pertuzumab Fab and the binary complex elutes earlier than HER2 alone. The purified complex is further complexed with trastuzumab Fab and the ternary complex elutes earlier than the dimer. The UV absorption is normalized.

Previous computational and biophysical studies have demonstrated that both trastuzumab Fab and pertuzumab Fab could bind simultaneously on HER2 ECD.[9, 10] To obtain the ternary complex of HER2 with both Fabs, we purified the binary complex of HER2 with the first Fab using size exclusion chromatography (SEC) before adding the second Fab. Based on the SEC profiles, HER2 formed stable binary complex with either Fab in solution and no HER2 peak was observed when excessive amount of Fab was present (Fig 1). The addition of the second Fab clearly shifted the elution volume again in SEC, indicating the presence of the larger ternary complex, and there was no obvious HER2-Fab binary complex detected as a shoulder peak (Fig 1). This experiment also demonstrated that the order of Fab binding doesn’t affect the ternary complex formation.

**Fig 2:**
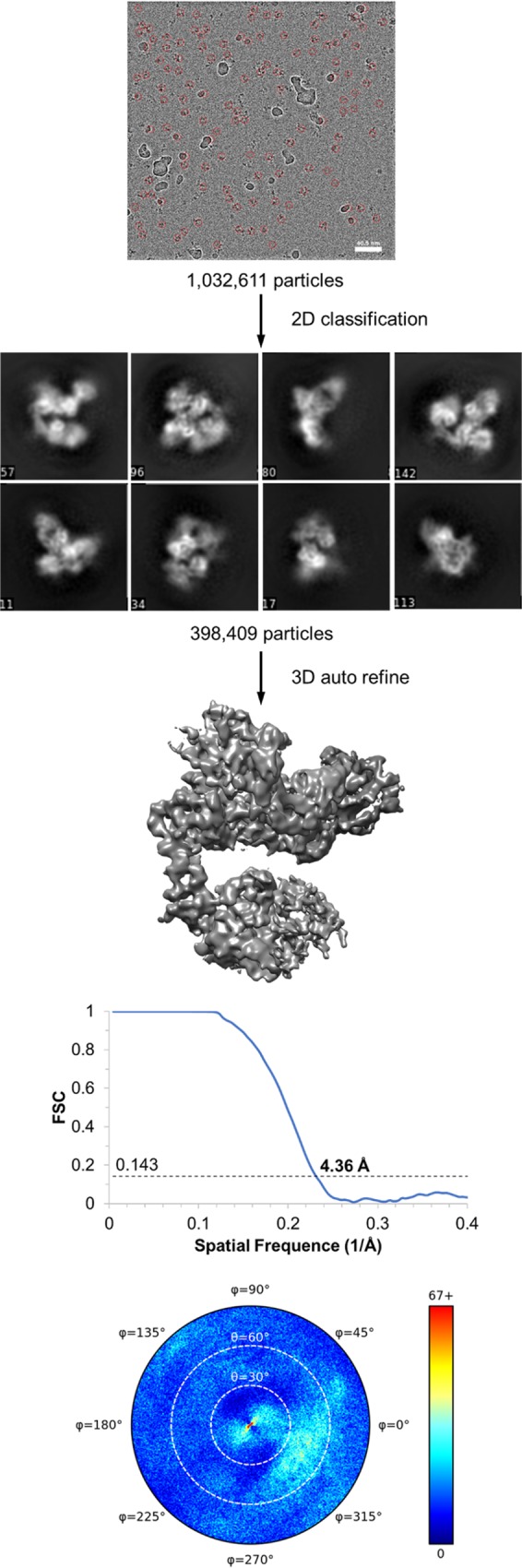
Brief summary of cryo-EM data processing. 1,032,611 particles were picked automatically and extracted in *cis*TEM software [28]. Three rounds of 2D classification were carried out and classes with clearer structure features were selected. Representative classes are shown here. A total of 398,409 particles were used in 3D Auto Refine and the final reconstruction was generated. The resolution was determined with FSC cutoff at 0.143.

**Fig 3:**
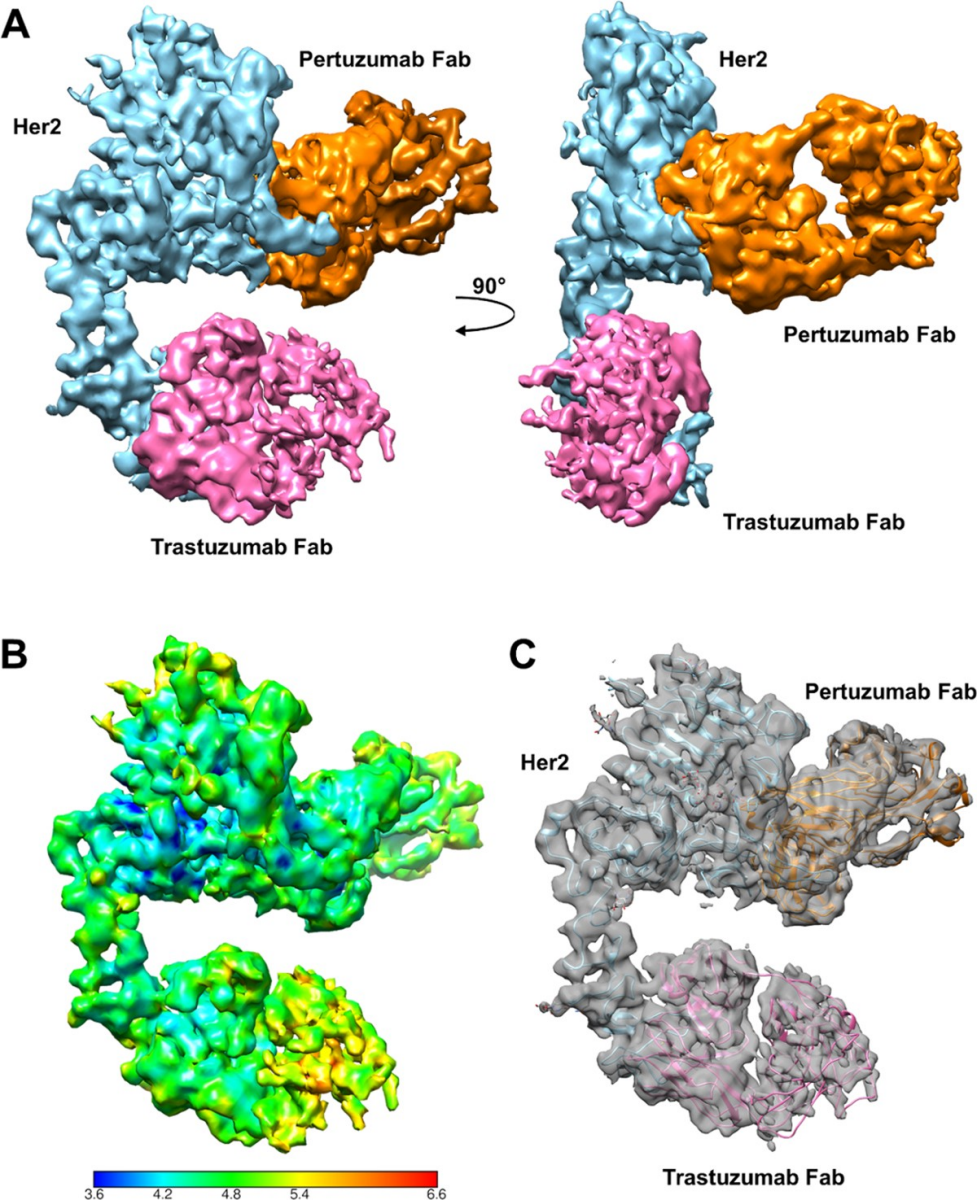
Cryo-EM map of HER2-trastuzumab-pertuzumab. A. Cryo-EM map showing the three components of the ternary complex: HER2 (sky blue), trastuzumab Fab (pink), and pertuzumab Fab (orange). B. Local resolution analysis of the final construction calculated using ResMap. C. Final construction (grey surface) with HER2 (sky blue), trastuzumab Fab (pink), and pertuzumab Fab (orange) fitted in the map. Glycans on HER2 are shown as sticks.

The purified ternary complex of HER2-trastuzumab-pertuzumab was subject to structural characterization using cryo-EM, and a density map was obtained at a global resolution of 4.36 Å (Fig 2). All three components of the ternary complex were identified in the cryo-EM map (Fig 3), and a final model of HER2-trastuzumab-pertuzumab was built and refined. Residues Thr23-Ala644 of HER2 were defined in the cryo-EM map except T127-V129 contained within a loop region. Predicted glycosylation on residues Asn68, Asn187, Asn259 and Asn571 could be assigned. Both trastuzumab Fab (light chain residues Asp1-Cys214 and heavy chain residues Glu1-Pro220) and pertuzumab Fab (light chain residues Asp1-Cys214 and heavy chain residues Glu1-Cys216) could also be fitted into the map. However, the constant region of trastuzumab Fab has poor density compared to its variable region and the other two components of the complex (Fig 3C), indicating its flexibility in solution. This is also consistent with the local resolution analysis using ResMap[11], which shows relative low resolution of this region (Fig 3B).

**Fig 4:**
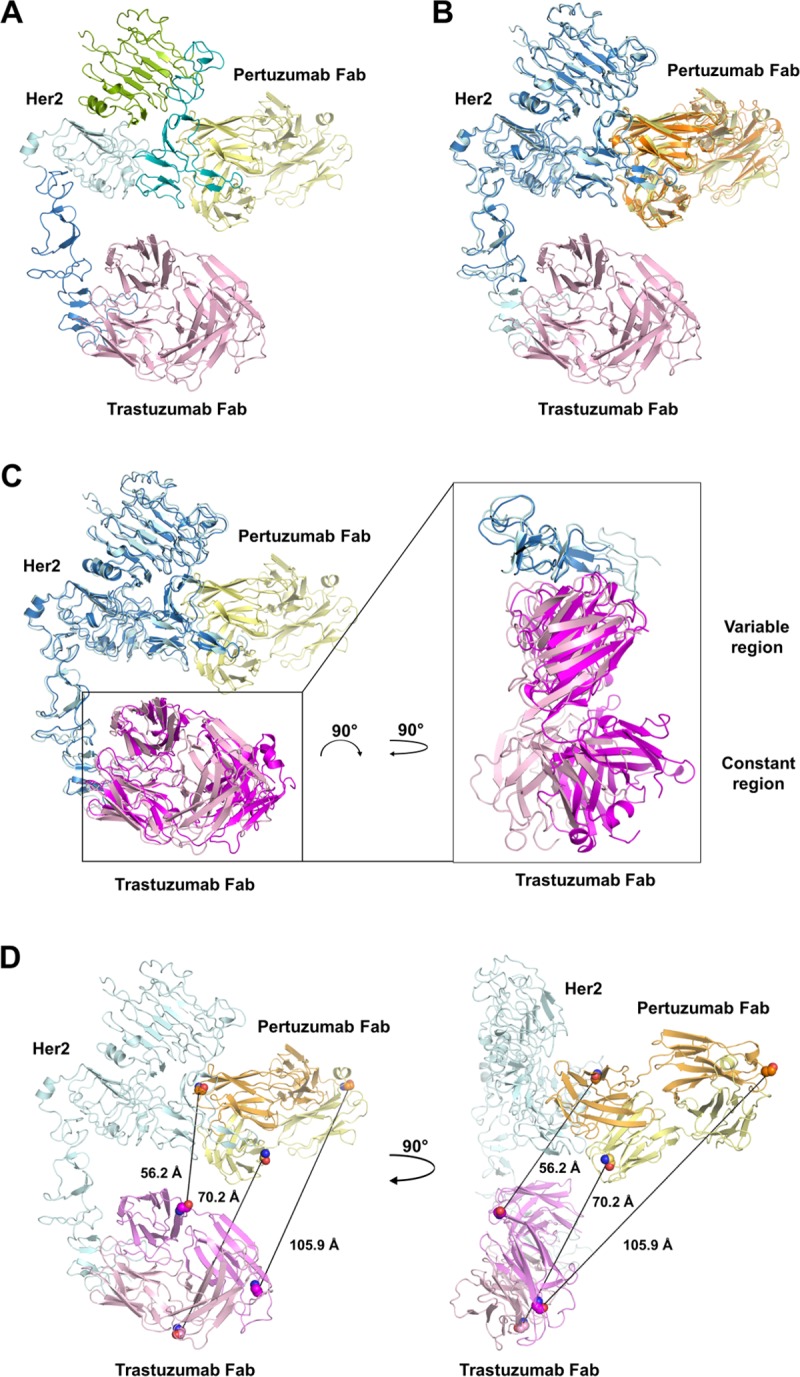
Overall structure of HER2-trastuzumab-pertuzumab and comparison with HER2-pertuzumab (PDB: 1S78) and HER2-trastuzumab (PDB: 1N8Z) structures. A. Overall structure of HER2-trastuzumab-pertuzumab. Domain I through domain IV of HER2 are shown in green, teal, cyan, and blue respectively. Trastuzumab Fab and pertuzumab Fab are shown in pink and yellow respectively. The previously proposed pertuzumab-induced new trastuzumab epitope is highlighted in magenta. B. Structure comparison of HER2-trastuzumab-pertuzumab and HER2-pertuzumab. In HER2-trastuzumab-pertuzumab, HER2, pertuzumab and trastuzumab are shown in cyan, yellow and pink respectively. In HER2-pertuzumab, HER2 and pertuzumab are shown in blue and orange respectively. C. Structure comparison of HER2-trastuzumab-pertuzumab and HER2-trastuzumab. In HER2-trastuzumab-pertuzumab, HER2, trastuzumab and pertuzumab are shown in cyan, pink and yellow respectively. In HER2- trastuzumab, HER2 and trastuzumab are shown in blue and magenta respectively. D. Distances between the C-terminus of trastuzumab V_L_ and N-terminus of pertuzumab V_L_ (68.6 Å), between C-terminus of trastuzumab V_H_ and N-terminus of pertuzumab V_H_ (56.2 Å), and between the C-termini of trastuzumab C_H_1 and pertuzumab C_H_1 (99.7 Å) were measured. The residues used for measurement are highlighted in spheres. Light chain and heavy chain of trastuzumab Fab are shown in pink and magenta. Light chain and heavy chain of pertuzumab Fab are shown in yellow and orange. The sequences used as nine-residue linkers in TP_L_ bispecific antibody are highlighted in teal (light chain) and blue (heavy chain).

The cryo-EM structure of HER2-trastuzumab-pertuzumab (Fig 4A) superimposes well with the crystal structures of HER2-pertuzumab (RMSD of 0.96 Å over 790 Cα atoms, Fig 4B) and HER2-trastuzumab (RMSD of 1.33 Å over 542 Cα atoms, Fig 4C). HER2 in the ternary complex adopts the same conformation as in the binary complexes and apo.[12, 13] Pertuzumab Fab in HER2-trastuzumab-pertuzumab aligns well with that in HER2-pertuzumab including the constant region (Fig 4B). In comparison, the variable region of trastuzumab Fab in HER2-trastuzumab-pertuzumab superimposes well with that in HER2-trastuzumab while its constant region is shifted from that in HER2-trastuzumab (Fig 4C). This is likely because the trastuzumab Fab, particularly its constant region, in the HER2-trastuzumab structure is involved in extensive crystal contacts whereas the pertuzumab Fab in the HER2-pertuzumab structure is not.[12, 13] Importantly, the interactions between HER2 and the two Fabs are very similar among the ternary and binary complexes[12, 13] with pertuzumab and trastuzumab Fabs bound to domains II and IV of HER2 respectively. Comparison of the cryo-EM structure of HER2-trastuzumab-pertuzumab with the crystal structures of HER2-pertuzumab and HER2-trastuzumab reveals that both pertuzumab and trastuzumab can bind to HER2 simultaneously with little conformational change and suggests that binding of one antibody does not enhance the binding of the other, in good agreement with previous biophysical studies.[10] Therefore, the clinical synergism of pertuzumab and trastuzumab likely arises not from enhanced affinity but from other mechanisms including synergy in the inhibition of HER2 ligand-dependent and ligand-independent signaling.

**Fig 5:**
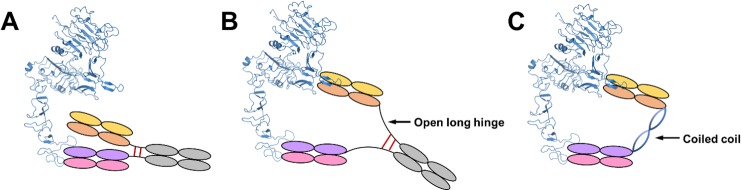
Schematic representation of bispecific molecules targeting HER2. A. Bispecific antibody with knob-into-hole Fc. B. Bispecific molecules with engineered hinge region of IgG3. C. Bispecific molecules connected by a dimeric coiled coil.

Since the combination treatment of trastuzumab and pertuzumab demonstrated superior inhibitory effect on the survival of HER2-positive breast cancer cells *in vitro*, *in vivo* and in clinical trials,[14–17] various bispecific antibodies containing both trastuzumab and pertuzumab variable regions were designed and evaluated. The first approach was the tetravalent “dual-variable-domain immunoglobulin”,[18] by fusing the variable domains of light chain and heavy chain (V_L_ and V_H_) of pertuzumab to the C-termini of the V_L_ and V_H_ of trastuzumab respectively, through nine-residue linkers derived from the constant domains (C_L_ and C_H_1) of trastuzumab.[19] One of the bispecific antibodies, TP_L,_ recognized the same epitopes as trastuzumab and pertuzumab, potently inhibited the *in vitro* HER2 heterodimerization and signaling, and suppressed the *in vivo* growth of breast tumor xenografts.[19] The second approach was the knob-into-hole Fc technique, in which half antibodies of trastuzumab and pertuzumab were assembled together.[20, 21] Strong inhibition of HER2-positive breast cancer cell proliferation was also observed *in vitro* and *in vivo* for these bispecific antibodies KN026 and MBS301.[20, 21] However, the anti-tumor activities of all the bispecific antibodies from both approaches were comparable to or slightly better than the combination of trastuzumab and pertuzumab. It appears from the cryo-EM structure of HER2-trastuzumab-pertuzumab that, even with the flexibility of the hinge region and the flexibility between the variable and the constant regions, the two Fab arms of one bispecific antibody cannot bind to both domains II and IV of one HER2 molecule simultaneously (Figs 4D and 5A), considering all the various conformations of IgG1.[22] This is consistent with the similar binding affinities of these bispecific antibodies for HER2 to those of trastuzumab and pertuzumab as well as their Fabs.[21, 23] In contrast, a bispecific molecule with variable regions engaging both domains II and IV of one HER2 molecule concurrently would have a substantially higher affinity, possibly as high as the product of the binding affinities of trastuzumab and pertuzumab. Such bispecific antibodies could be developed by introducing an engineered hinge region of IgG3 to increase the Fab domain flexibility necessary for hetero-bivalent binding to HER2 (Fig 5B). Similar bispecific anti-HIV antibodies have shown synergistic potent activity.[24] Such bispecific molecules can also be engineered by connecting Fab regions engaging both domains II and IV of HER2 to a rigid protein linker (such as a dimeric coiled coil) with optimal length (about 100 Å) (Fig 5C) and these hetero-diFabs could have anti-tumor activities far superior to the combination of trastuzumab and pertuzumab. As a case in point, similar optimal hetero-diFabs designed against the HIV envelope trimer have exhibited up to 2.5 orders of magnitude increased potency.[25]

## Materials and methods

### Protein purification and complex formation

Trastuzumab (Herceptin) and pertuzumab (Perjeta) were purchased from Genentech. Each antibody was cleaved with papain at 37° for 6 h in a solution containing 20 mM tris pH 8.0, 150 mM NaCl, 1 mM EDTA, 20 mM cysteine, 1 mg/mL antibody and 0.01 mg/mL papain. The reaction was quenched by adding 25 mM iodoacetamide. The Fab fragment was separated from Fc fragment by cation exchange chromatography at pH 4.0. Purified Fab was subject to size exclusion chromatography with Superdex 200 10/300 column (GE Healthcare) in 20 mM HEPES pH 7.5, 150 mM NaCl (buffer A). Lyophilized human HER2 (ACRO Biosystems) was reconstituted and incubated with an excess of the first Fab. The complex was purified with size exclusion chromatography and subsequently incubated with an excess of the second Fab. The ternary complex containing HER2 and both trastuzumab Fab and pertuzumab Fab was further separated from the unbound second Fab by size exclusion chromatography in buffer A.

### Cryo-EM sample preparation and data acquisition

For cryo-EM, 3 μl of HER2-trastuzumab-pertuzumab at 2.4 mg/ml was applied to a glow-discharged Quantifoil R1.2/1.3 300 mesh grid. The sample was then vitrified with a FEI Vitrobot Mark IV at 100% humidity using 2.5 sec blot time. Cryo-EM data was acquired at 300 kV on a FEI Titan Krios. Dose fractionated movie frames were collected at 130,000x nominal magnification (corresponding to a physical pixel size of 1.059 Å) on a K2 summit direct electron detector (Gatan). A total of 6 seconds exposure with 0.2 second subframes were recorded in superresolution counting mode with a total dose of 45 electrons per Å^2^.

### Cryo-EM data processing and model building

Movies of HER2-trastuzumab-pertuzumab complex were dose-weighted and corrected for beam-induced motion using Unblur.[26] CTF estimation was done with CTFFIND4[27] using a resolution range of 30–4 Å. Micrographs with fit resolution worse than 10 Å were not included in the subsequent processing. 1,032,611 particles were picked automatically using a low-pass filtered disk with a characteristic radius of 55 Å at a threshold of 3.0 in *cis*TEM software[28]. The particles were extracted with a box size of 208 × 208 pixels and underwent three rounds of 2D classification into 200 classes with a mask radius of 78 Å. C1 symmetry was imposed during processing. Classes with clearer structure features were selected and a total of 398,409 particles were subject to 3D Auto Refine in *cis*TEM[28] in a single 3D class with a starting resolution limit of 30 Å. The initial reference map was generated with the Ab-Initio 3D function in *cis*TEM and low-pass filtered for 3D refinement. The final refined map with a global resolution of 4.36 Å was sharpened with Phenix.autosharpen by applying a B-factor of 254.43 Å^2^. The initial model of HER2-trastuzumab-pertuzumab complex based on the crystal structures of HER2-trastuzumab (PDB: 1N8Z) and HER2-pertuzumab (PDB: 1S78) was fitted into the cryo-EM map in COOT[29] and briefly rigid-body refined. Glycans and missing loops were manually built with the map displayed at σ around 3.5 or 4. Several rounds of manual adjustment in COOT[29] and refinement using Phenix.real_space_refine[30] were carried out to achieve the final structure. Refinement process was monitored with MolProbity.[31] Structure figures were generated using PyMOL (Schrödinger, LLC.) and UCSF Chimera.[32] The cryo-EM map and coordinates of HER2-trastuzumab-pertuzumab structure have been deposited in the electron microscopy data bank with access number EMD-7137 and in the Protein Data Bank with access code 6OGE respectively.
