# Correction: Cryo-EM Structure of HER2-trastuzumab-pertuzumab complex

**DOI:** 10.1371/journal.pone.0217716

**Published:** 2019-05-28

**Authors:** Yue Hao, Xinchao Yu, Yonghong Bai, Helen J. McBride, Xin Huang

In the Materials and methods section, the access number for the electron microscopy data bank is incorrect. The correct access number is: EMD-20055.
